# Supplementary material for: Discovery and application of insertion-deletion (INDEL) polymorphisms for QTL mapping of early life-history traits in Atlantic salmon
Source: BMC Genomics. 2010 Mar 8;11:156. doi: 10.1186/1471-2164-11-156 (PMC2838853; doi:10.1186/1471-2164-11-156)
Supplement: Additional file 2 — Information on developed 76 locus single-run INDEL panel in Atlantic salmon. Information on fluorescence labeling, primer concentrations, PCR pooling and links to alignments, INDEL motifs and GENESCAN (Burge and Karlin 1997) predictions of genes/exons are available in html format. [file 1471-2164-11-156-S2.ZIP › Additionalfile2/snpsummary8921.html]

```
Cluster 2859 Contig 2

prev  Summary    Contig List  next
```

Size of Consensus sequence = 690

Number of sequences = 6

Minimum redundancy = 2

Key

A gi|85033971|gb|DW562627.1|DW562627 EST\_ssal\_rgb2\_27046 rgb2 Salmo salar cDNA clone ssal\_rgb2\_543\_368\_fwd 3', mRNA sequence  
B gi|24384513|gb|CA054270.1|CA054270 ssalrgb517150 mixed\_tissue Salmo salar cDNA, mRNA sequence  
C gi|85029282|gb|DW557938.1|DW557938 EST\_ssal\_rgb2\_22357 rgb2 Salmo salar cDNA clone ssal\_rgb2\_536\_136\_fwd 3', mRNA sequence  
D gi|85033479|gb|DW562135.1|DW562135 EST\_ssal\_rgb2\_26554 rgb2 Salmo salar cDNA clone ssal\_rgb2\_543\_050\_fwd 3', mRNA sequence  
E gi|85050362|gb|DW578540.1|DW578540 EST\_ssal\_rgb2\_42959 rgb2 Salmo salar cDNA clone ssal\_rgb2\_569\_243\_fwd 3', mRNA sequence  
F gi|89844989|gb|DY701112.1|DY701112 EST\_ssal\_rgb2\_56851 ssalrgb2 mixed\_tissue Salmo salar cDNA Salmo salar cDNA clone ssal\_rgb2\_591\_311\_fwd 3', mRNA sequence

3 SNPs detected

A B C D E F  cosegregation weighted

158 T C C C T C   1/3 33.33
369 T T T - T -   2/3 66.67
370 G G G - G -   2/3 66.67
